# Uptake of a self‐guided digital treatment for depression and anxiety: A qualitative study exploring patient perspectives and decision‐making

**DOI:** 10.1111/hex.13976

**Published:** 2024-01-19

**Authors:** Alana Fisher, Sylvia Eugene Dit Rochesson, Madelyne A. Bisby, Amelia J. Scott, Milena Gandy, Andreea Heriseanu, Nick Titov, Blake Dear

**Affiliations:** ^1^ The eCentreClinic Macquarie University Sydney New South Wales Australia; ^2^ The MindSpot Clinic MQ Health Sydney New South Wales Australia

**Keywords:** anxiety, decision‐making, depression, digital treatment, iCBT, patient perspectives, qualitative

## Abstract

**Background:**

Despite the demonstrated efficacy and potential scalability of self‐guided digital treatments for common mental health conditions, there is substantial variability in their uptake and engagement. This study explored the decision‐making processes, influences and support needs of people taking up a self‐guided digital treatment for anxiety and/or depression.

**Methods:**

Australian‐based adults (*n* = 20) were purposively sampled from a trial of self‐guided digital mental health treatment. One‐to‐one, semistructured interviews were conducted, based on the Ottawa Decision‐Support Framework. Interviews were transcribed verbatim and analysed thematically using framework methods. Baseline sociodemographic, clinical and decision‐making characteristics were also collected.

**Results:**

Analyses yielded four themes. Theme 1 captured participants' openness to try self‐guided digital treatment, despite limited deliberation on potential downsides or alternative options. Theme 2 highlighted that immediacy and ease of access were major drivers of uptake, which participants contrasted with gaps in access and continuity of care in face‐to‐face services, especially rurally. Theme 3 centred on participants as the main agents in their decision‐making, with family and health professional attitudes also reportedly influencing decision‐making. Theme 4 revealed participants' primary motivations for deciding to take up treatment (e.g., the potential to increase insight and coping skills), while also acknowledging that pre‐existing characteristics (e.g., health and digital literacy, insight) determined participants' personal suitability for self‐guided digital treatment.

**Conclusion:**

Findings help to elucidate the decision‐making influences and processes amongst people who started a self‐guided treatment for depression and anxiety. Additional information and decision support resources appear warranted, which may also improve the accessibility of self‐guided treatments.

**Public or Patient Contribution:**

Patients were interviewed about their views and experiences of decision‐making about accessing and taking up treatment. As such, patient contribution to the research was as study participants.

## 1 INTRODUCTION

Mental health conditions affect almost one in three people worldwide over their lifetime,^1^ with depression and anxiety being the most common^2^, ^3^ and contributing significantly to the global burden of disease.^4^, ^5^ Despite this, even in high‐income countries, the majority of people do not access professional help or treatment for their mental health^6^, ^7^ and of those who do, only a minority receive evidence‐based or minimally adequate levels of care^8^, ^9^ With the view to increasing access to treatment, internet‐delivered treatments overcome many of the practical and attitudinal barriers associated with accessing traditional face‐to‐face treatment for mental health^10^, ^11^ through their low‐or‐no‐cost, discreet, flexible, accessible and scalable delivery with minimal delays or waitlists. Importantly, digital mental health treatments such as Internet‐delivered cognitive behavioural therapy (iCBT) with therapist support have been found to be as effective as face‐to‐face CBT.^12^, ^13^

Digital mental health treatments may be offered in unguided or guided formats.^14^ Although there is some variability, guided formats include support and guidance from a trained mental health professional (therapist or clinician), in the form of phone calls, videoconferencing, emails or private messages.^15^, ^16^ Unguided formats are completed independently, or with limited provision of technical and administrative support by nonclinical personnel, such as a research assistant or other clinic support staff.^15^, ^16^ In this way, self‐guided digital mental health treatments may have greater scalability and cost‐effectiveness potential than guided treatments, while still being highly acceptable, safe and effective at improving symptoms of depression and anxiety.^17^, ^18^

However, despite their promise, a recent meta‐analysis of randomised‐controlled trials (RCTs)^19^ has demonstrated that people accessing completely self‐guided digital treatments for depression (i.e., no human contact before or during the intervention) show less symptom improvement posttreatment, lower adherence and higher drop‐out compared to people accessing fully guided digital treatments (i.e., human contact before and during the intervention). In light of this evidence, and the reported link between patient engagement in healthcare decision‐making and later adherence to treatment plans,^20^ it is important to understand people's decision‐making around which treatment format they feel will be most appropriate and beneficial for them based on the evidence, realistic expectations of treatment, personal preferences and life circumstances.

Critically, people can already access digital mental health treatments within routine care and without needing a referral. In Australia, these include MindSpot (mindspot.org.au) and ThisWayUp (thiswayup.org.au), while people overseas can freely access MoodGym (moodgym.com.au) and MyCompass (mycompass.com.au). While some treatments are therapist‐guided^21^, ^22^, others are self‐guided without guidance or support from a trained health professional either before or during the intervention.^23^, ^24^ Again, this absence of clinician guidance means that there is a need to ensure that resources and processes are in place to support people to make informed treatment decisions independently. Better understanding the decisional needs of people considering self‐guided digital treatment may also allow treatment providers to anticipate and better address the patient‐reported barriers to later ongoing engagement in treatment, such as finding motivation, perceiving treatment as useful and integrating treatments into daily routines.^25^, ^26^

The Ottawa Decision‐Support Framework (ODSF) is one model that represents factors that are likely to lead to high‐quality social and healthcare decisions.^25^, ^27^, ^29^ According to the ODSF, the quality of decisions is influenced by people's (i) knowledge of options, (ii) expectations and values about outcomes, (iii) perceptions about the opinions and practices of others, (iv) decisional conflict (i.e., low personal uncertainty about the best course of action) and (v) availability of support and resources to make and action decisions.^27^, ^28^, ^29^ When these five factors are addressed in decision‐making, people are more likely to make a high‐quality decision, that is, the person is informed about, has realistic expectations and is clear on their values and personal preferences with regard to the available options and outcomes, is well supported by others, has low decisional conflict and has access to resources to help them implement their decision. The ODSF model also proposes that individuals are likely to encounter challenges in making decisions that align with personal preferences without adequate information or support, or when viable options are available, as is the case with mental health treatments. As such, applying the ODSF to decision‐making about self‐guided digital treatments may elucidate the most salient influences in a mental health context that are likely to enable or hamper high‐quality decisions about treatment uptake.

To this end, the current study aimed to explore the views and experiences of people who had decided to take up self‐guided digital mental health treatment. Specifically, this study aimed to elucidate personal perspectives on (i) decision‐making processes, (ii) influences on decision‐making and (iii) decisional needs.

## 2 METHODS

### 2.1 Participants

Participants were recruited from an international RCT evaluating the impacts of health‐related beliefs and support needs on uptake and engagement with the Wellbeing Course—a transdiagnostic iCBT treatment for people with symptoms of anxiety and/or depression (see Section 2.2 below).

To be eligible for the overall trial, participants were required to be (i) an adult (≥18 years), (ii) with reliable internet and computer access, (iii) able to read and understand English, (iv) self‐identifying as having symptoms of anxiety and/or depression and (v) at no immediate risk of self‐harm or suicide.

Additionally, participants were only eligible for the interview substudy if they were residing in Australia and had a Medicare card (i.e., Australia's universal health insurance scheme, which allows residents and some overseas visitors access to a wide range of health and hospital services at low or no cost). Participants were purposely selected to ensure a mix of background characteristics (e.g., age, gender, education level and experience with mental health help‐seeking) that were likely to yield a diverse range of perspectives and experiences. For example, in certain phases of the RCT, only male or younger‐age trial participants were invited to participate in an interview, because they were underrepresented in the sample at that time (see Section 2.3 below). Participants were recruited until data saturation, whereby no new themes or subthemes were evident in interviews.^30^

### 2.2 Treatment and context

Participants registered their interest for the Wellbeing Course via the eCentreClinic (https://www.ecentreclinic.org/), a specialist research unit and not‐for‐profit initiative of Macquarie University, which provides people with access to free online psychological treatment through participation in research trials. Upon landing on the clinic homepage, participants navigate to their treatment or ‘course’ of interest, where they will find brief information outlining what treatment involves, who it is suitable for and the focus of the current research trial.

The Wellbeing Course (https://www.ecentreclinic.org/wellbeing-course) comprises 5 weekly modules or ‘lessons’ (comprising core CBT components, such as psychoeducation, cognitive restructuring, behavioural activation, graded exposure and relapse prevention) delivered over eight consecutive weeks, as well as case studies based on previous participants, ‘DIY’ homework‐style exercises and additional resources targeting skill areas (e.g., assertive communication, management of sleeping difficulties).

The current RCT offered the self‐guided (unguided) version of the course without any clinical interview/assessment over the phone with a clinician. Instead, participants’ suitability and eligibility for the course were determined by a clinical researcher (AF), who reviewed participant responses to an initial screening and assessment survey. Once the course has started, participants receive automated email reminders to log in and complete the lessons, are monitored for risk and symptom deterioration and can email the digital clinic team (contact@ecentreclinic.org) if they experience technical difficulties. Several trials have demonstrated that the Wellbeing Course is safe and clinically efficacious in the treatment of depression and anxiety in both the therapist‐guided and self‐guided versions.^18^, ^31^, ^32^, ^33^, ^34^

### 2.3 Procedure

This research received ethical approval from Macquarie University's Human Research Ethics Committee (ref: 520211080734189). Eligible participants were emailed the week before commencing treatment, inviting them to take part in a one‐to‐one phone interview. Interested participants were asked to review the attached information and consent form and reply with their availability for an interview. The lead researcher (A. F.) then arranged for interviews to be conducted with participants in the days before them commencing treatment. Participants were not offered any incentives to be interviewed.

### 2.4 Qualitative data collection

Interviews were conducted by researchers (AF and SEDR) with experience in conducting semistructured interviews within the field of clinical and health psychology. All interviews were audio‐recorded, transcribed using an online speech to text transcription software (otter.ai) and checked for accuracy by members of the research team.

A semistructured interview protocol was purpose‐designed for this study, based on the decisional needs described within the ODSF.^27^, ^28^, ^29^ A combination of open‐ended questions and follow‐up prompts asked participants to reflect on their decision to take up self‐guided digital mental health treatment (i.e., Wellbeing Course), including their awareness and knowledge of alternative options for mental health support, expectations for treatment, deliberation with important others (e.g., family and/or health professional) and any personal uncertainty with their decision. Participants were also asked to comment on any information or resources that they would have wanted to support their decision‐making about treatment.

### 2.5 Quantitative data collection

Quantitative data were collected when participants completed the initial screening and assessment survey for the RCT. To characterise the sample and ensure a diversity of background characteristics, we included a series of validated and purpose‐designed self‐report measures asking participants for their sociodemographic characteristics (e.g., age, gender, education level, etc.), past and current help and treatment seeking for their mental health, decisional conflict and symptoms of depression and anxiety. Validated measures are described below:

*Patient Health Questionnaire 9‐Item Scale* ^35^ measures depression symptoms; scores range from 0 to 27. Higher scores correspond to greater symptom severity, and scores of ≥10 are considered indicative of major depressive disorder, although recent studies indicate that higher cut‐offs may be appropriate.^36^

*Generalized Anxiety Disorder 7‐Item Scale* ^37^ measures anxiety symptoms; scores range from 0 to 21, with higher scores corresponding to greater symptom severity, and scores of ≥10 indicative of generalised anxiety disorder.

*Decisional Conflict Scale* (*DCS*)^38^, ^39^ 10‐item scale is designed to assess people's perceived difficulties in making a healthcare decision and was adapted from the original 16‐item scale to be suitable for lower‐literacy populations. This scale format includes four subscales of feeling: (i) uninformed, (ii) unsupported in decision‐making, (iii) unclear about their personal values and (iv) uncertain about the best course of action. Each item is rated either ‘Yes’, ‘Unsure’ or ‘No’, with ratings then converted into a score out of 100 for each subscale, and for the total score. In all instances, higher scores are indicative of higher levels of decisional conflict.

### 2.6 Data analysis

Interviews were analysed thematically using framework methods described by Ritchie et al.,^40^ which involve five phases: (1) Familiarisation with the data; (2) creating a thematic framework; (3) indexing; (4) charting and (5) mapping and interpretation. See Table 1 for a summary of each analysis phase. A thematic analysis approach was chosen because of the flexibility that it affords researchers, to analyse qualitative data both inductively (i.e., driven by the data itself) as well as deductively (i.e., based on existing empirical literature and conceptual frameworks, including constructs within the ODSF).^27^, ^28^, ^29^

**Table 1:** Description of thematic analysis phases using framework methods.

**Phase**	**Description**
1) *Familiarisation with the data*	Members of the research team (A. F. and S. E. D. R.) read the interview transcripts as part of accuracy checking with the audio recording and while developing the thematic framework.
2) *Creating a thematic framework*	Three interview transcripts with participants from different demographic backgrounds were independently read and coded by two researchers (A. F. and S. E. D. R.). Coding involved assigning a descriptive label to relevant phrases or sentences using the comment function in Microsoft Word. Afterwards, the researchers met to discuss and compare their coding and interpretations of the data. These discussions were used to develop a coding framework, in which the codes were organised hierarchically into themes and subthemes based on common patterns of meaning in the data.
3) *Indexing*	All remaining interview transcripts were coded by the researchers (A. F. and S. E. D. R.) using the preliminary coding framework in NVivo. Throughout this process, the researchers met weekly to discuss their interpretations of the data and changes to the coding framework. Changes occurred when codes were developed to account for new meaningful phrases in the interview transcripts and when codes were collapsed together or edited to better reflect their common pattern of meaning.
4) *Charting*	To assist interpretation of the data, coded interview data were charted into Microsoft Excel by S. E. D. R.^41^ In this framework matrix, participants were represented by rows and themes by columns, with the coded data added to their intersecting cells.
5) *Mapping and Interpretation*	The lead researcher (A. F.) used the framework matrix to identify patterns and relationships within the coded data. These patterns reflected generalised experiences across all participants as well as similarities or differences within and between participants' experiences.

Quantitative data were analysed descriptively using IBM SPSS Statistics 29 to obtain frequencies for categorical variables and means and standard deviations for continuous variables.

## 3 RESULTS

### 3.1 Participant characteristics

Demographic and background clinical characteristics of the 20 participants are presented in Table 2.

**Table 2:** Background characteristics of participants (*n* = 20).

	*M* (SD; min–max)
Age	51 (11.95; 23–69)
GAD‐7 (anxiety /21)	9.75 (4.03; 3–17)
PHQ‐9 (depression /27)	12.45 (4.90; 5–20)
DCS‐10 (decisional conflict /100)	
Uncertainty	11.84 (21.03; 0–50.00)
Informed	18.42 (19.16; 0–66.67)
Values clarity	15.79 (20.77; 0–50.00)
Support	11.40 (15.77; 0–50.00)
Total score	14.47 (13.83; 0–45.00)

	*n* (%)
Gender	
Female/woman	15 (75)
Male/man	5 (25)
Locality	
Urban or metropolitan	12 (60)
Regional or remote	8 (40)
Highest education	
Year 12/grade 12	1 (5)
Associate diploma/apprenticeship	2 (10)
Undergraduate degree	12 (60)
Postgraduate degree	5 (25)
Relationship status	
Married/de facto	12 (60)
Divorced/separated	4 (20)
Single/dating	3 (15)
Widowed	1 (5)
Seen GP for mental health (yes)	16 (80)
Seen other health professionals for mental health (yes)	15 (75)
Never seen a health professional for mental health	4 (20)
Ever taken medication for mental health (yes)	12 (60)
Current medication for mental health (yes)	8 (40)
Ever received psychological support (yes)	13 (65)
Current (other) psychological support (yes)	8 (40)
Duration of anxiety/depression	
More than 2 weeks, less than 6 months	1 (5)/2 (10)
More than 6 months, less than 1 year	2 (10)/2 (10)
1–5 years	6 (30)/5 (25)
More than 5 years, less than 10 years	2 (10)/2 (10)
More than 10 years	7 (35)/6 (30)
Not applicable	2 (10)/3 (15)

Abbreviations: DCS‐10, Decisional Conflict Scale 10‐Item Scale; GAD‐7, Generalized Anxiety Disorder 7‐Item Scale; GP, general practitioner; PHQ‐9, Patient Health Questionnaire 9‐Item Scale.

### 3.2 Qualitative findings

Qualitative analyses identified four overarching themes, which are summarised in the following sections. See Table 3 for illustrative participant quotes, arranged according to theme. Corresponding participant IDs are cited in text.

**Table 3:** Illustrative participant quotes relating to each theme.

**Themes**	**Participant quotes**
Theme 1: *Navigating treatment options: focussing mainly on the positives* This theme centred on patients' attitudes towards digital treatments for health issues. It captured participants’ general willingness to try digital treatments, influenced by perceived low risks and positive past experiences with similar treatments.	‘I thought [that] this [digital treatment] has got to be good… if it doesn't work for me then I've lost nothing… but I know from my previous dealings with psychology [that] it's going to benefit me’. (ID4) ‘…the information you provided was good…the questions (FAQs) were good. It [the uptake process] was all very seamless and easy… I don't think there'd [need to be] further information’. (ID15) ‘…[If] I could talk to someone [at the eCentreClinic] there and then or have someone call me to talk about the [treatment] options, that would have been handy…. It would have been helpful if it summarised what options to take’. (ID 3)
Theme 2: *Ease of access and credibility* This theme focussed on the perceived accessibility, flexibility and trustworthiness of digital treatments; it emphasised the adaptability of these treatments to diverse mental health needs as well as their credibility within endorsements from reputable sources and assurances of confidentiality.	‘…at one point I was looking for [in‐person] appointments with therapist or counsellor, but they all cost quite a lot. I don't think that I can afford private therapy sessions’. (ID19) ‘…[this digital treatment is] more general and it would help me with my general wellbeing, because there might be some problems that I am not aware of yet… it will help me improve a lot of things at once…’ (ID19) ‘…there would be strategies [in the course] to help me to maintain good mental health because of [having] a history of relapsing’. (ID11) ‘…it's [the course is] actually run by health professionals and psychologists… although this is an online course… it's written by and managed by real people in the university setting’. (ID 6) ‘I had one psychologist [previously in person]who went on parental leave… I wanted my mental health to have consistent support. And I thought, “Okay, well, where else can I go? What else can I sort of do?” to get support…’ (ID1) ‘There are very few psychologists in town in the first place… I work with a lot of professionals who do psych[ological] treatment… I didn't really want to do anything face‐to‐face [in terms of treatments]… it would be hard for them [local psychologists] to offer me help in that way, and then have to work with me the next day on a professional level’. (ID1)
Theme 3: *Interactions with personal and health professional networks* This theme captured the integration of digital treatments with mainstream mental health services and personal networks; it highlighted participants’ desire for blended care models, the influence of healthcare professionals' attitudes and patients’ high levels of perceived autonomy when deciding to access digital treatments.	‘…it would be good if GPs had [digital treatments] as a tool in their toolbox to give to people that they felt they could benefit from it but don't need a full‐on intervention for their anxiety or depression… it could be a starting point say, 'Why don't you start with this for you?’ (ID9) ‘This was a personal decision [about accessing digital treatment] … that I thought was best for me’. (ID5) ‘…I just want to do something to make myself feel better and try to improve my self‐esteem. For me I don't need to talk about it [my mental health concerns] because it's a personal journey…’ (ID17)
Theme 4: *Personal characteristics needed to support uptake* This theme focussed on the intrinsic motivations and competencies required for individuals to engage with digital mental health treatments, such as the desire to acquire knowledge and practical skills, digital and health literacy and the ability to self‐assess motivation and suitability for self‐guided, structured digital treatments.	‘…the main thing is being able to develop my skills [with this digital treatment… with my current psychologist, I'm kind of done talking about the problem, if that makes sense…’ (ID1) ‘…for it [digital treatment] to be able to assist me to recover from depression and anxiety, to better manage my symptoms, and have a better understanding of my symptoms and how the skills can help with that’. (ID5). ‘…you need to be able to read and use a computer [to do digital treatment… but I think it's more about the intent, like the way that things are presented online’. (ID16) ‘The only thing I just don't know about is accountability [when doing digital treatment… Because it's self‐paced and me doing it myself. When I've got to check in with someone, I'm more likely to do it’. (ID16) ‘[the emails show] there's a team keeping track of what I'm doing… that motivates me to keep building habits. I like [treatment has] a start date, and an end date. I will spend like a specific amount of time to work on this. That will be helpful for me’. (ID19)

Abbreviation: GP, general practitioner.

#### 3.2.1 Theme 1: Navigating treatment options: Focussing mainly on the positives

Most participants expressed high levels of willingness and openness to trying digital treatments, in part because of their perceived lack of downsides and mentality of ‘I might as well give it a go…’ (ID1; see Table 1). Some participants cited positive past treatment experiences as influencing their attitudes and current decision‐making on uptake of digital treatment (ID4). By contrast, some other participants expressed apprehension regarding the level of clinician support offered as part of treatment (minimal and limited mainly to technical assistance).

In considering their uptake of self‐guided digital treatment, participants demonstrated limited knowledge and deliberation about alternative treatment options. The knowledge and awareness that participants did have mostly related to their experience of help‐seeking with a GP. Most commonly, participants reported seeing a GP to discuss (and subsequently take up) one‐on‐one (Medicare‐rebated or partly‐government subsidised) therapy with a psychologist or counsellor, or antidepressants.

Despite the limited research and information gathering reported by some participants, most acknowledged the role of information in supporting treatment decisions and promoting uptake. Participants also cited differing preferences for the content, amount and format of pretreatment information and decision support. Some participants expressed satisfaction (ID15). Meanwhile, a few other participants explained that they would have found it helpful to speak to someone to discuss options and ask questions (ID 3).

#### 3.2.2 Theme 2: Ease of access and credibility

This theme shed light on some of the key advantages of self‐guided digital treatments, as perceived by participants, including accessibility, flexibility in time and location, convenience, immediacy of treatment and low threshold to access, given minimum eligibility criteria. These advantages were said to motivate participants to take timely action on their symptoms of depression and anxiety while motivated, and therefore ‘strike while the iron is hot’ (ID 4).

Compared to traditional face‐to‐face treatments, digital treatments were also seen as easier to access because they are free of cost to the consumer (ID19, see Table 1). Participants also noted that although digital treatments were standardised in terms of their content and delivery, their transdiagnostic scope meant that they were well suited to a diverse range of symptom profiles (ID19). Further, participants felt that the information and skills presented in digital treatments could serve a dual purpose: as an introductory primer for first‐time help seekers or as a refresher for those with chronic and relapsing of depression and/or anxiety who had previously sought professional help for their symptoms' (ID11).

Trustworthiness and credibility were commonly cited by participants as essential factors when deciding where to seek help for their mental health online. Participants reported relying on credible third‐party resources when considering digital treatments, including mental health organisations, personal recommendations, patient stories and testimonials (appearing in online consumer forums). The digital service provision model via a research clinic, which included a team of qualified mental health professionals and research academics, also lent trustworthiness and credibility (ID 6).

Difficulties with access and disruptions to continuity of care in traditional (face‐to‐face) mental health services prompted participants to do self‐guided research online and be proactive in seeking digital treatments. For example, some participants researched online treatment options when encountering long waitlists, transitioning out of seeing a psychologist face‐to‐face and needing ongoing support, or when they felt a lack of therapeutic rapport (ID1).

Other participants, based in rural and remote areas, also mentioned that digital treatments not only addressed accessibility gaps due to a lack of face‐to‐face options but they also addressed issues of privacy and confidentiality that occurred when seeking help from local health professionals with whom they had pre‐existing professional or personal relationships (ID1).

#### 3.2.3 Theme 3: Interactions with personal and health professional networks

Participants spoke about the interaction between digital and mainstream face‐to‐face mental health services. Even though participants were accessing a self‐guided digital treatment, they expressed a desire for integrated and blended care models, such as having GPs refer them to digital services or use these services in conjunction with face‐to‐face therapy with a psychologist. Stepped care models, where self‐guided digital treatment represents the first step, were also alluded to by the participants (ID9; see Table 1).

Some participants spoke about dismissive or sceptical attitudes from GPs and other health providers towards digital or remotely delivered treatment options, which were sometimes ‘brushed off’ (ID9) or ‘scoffed at’ (ID1). These attitudes signalled to participants that they were receiving inadequate support, prompting them to engage in self‐guided research and information seeking about digital treatment options.

Levels of family involvement, attitudes towards help‐seeking and own experiences of help and treatment‐seeking all appeared to vary based on participant reports. Participants alluded to discussions with family and friends about mental health help‐seeking and support (or lack thereof) but did not link these discussions to their own decision‐making. Participants pointed out that the final decision to take up treatment was theirs and independent of family and friends (IDs 5 and 17). Some participants had spoken to family members about their decision to take up digital treatment; yet, participants were mostly unaware of their family members ever taking up digital treatment themselves.

#### 3.2.4 Theme 4: Personal characteristics needed to support uptake

When participants were asked to consider their motivations to take up self‐guided digital treatment, expectations of acquiring new knowledge and skills or building on existing knowledge and skills were highly valued. Participants preferred these expected treatment features over other types of counselling or psychotherapy (ID1, see Table 1). This was in part because participants thought that such knowledge and skills would aid their self‐management of depression and anxiety (ID5).

Participants noted that access to and uptake of digital mental health treatment relied on having adequate digital and health literacy. Participants felt that digital treatment providers needed to accommodate lower literacy levels, since literacy enables potential treatment seekers to evaluate available options, determine one's need for treatment and take up treatment with ease (ID16).

Participants spoke about the need to ‘self‐assess’ their levels of motivation and personal suitability for digital mental health treatment. Participants expressed ambivalence and uncertainty about the suitability of self‐guided digital treatment. Specifically, some participants felt that the self‐paced nature of this treatment required high levels of self‐motivation and discipline (ID16). Others, by contrast, saw the highly structured nature of this treatment as helping with motivation (ID19). Participants also explained that their decision to take up digital treatment was a response to there being limited alternative options (e.g., telephone support lines, face‐to‐face sessions with a private psychologist or counsellor, seeing their GP for information and support) and expressed uncertainty about their ability to cope if treatment involved ‘pushing through things [e.g., past trauma]’ (ID2) or ‘getting confronted with thoughts and feelings’ (ID20) without the support and guidance of a clinician.

## 4 DISCUSSION

This is the first known study to explore the decision‐making influences, processes and decisional needs among people taking up self‐guided digital treatment for depression and anxiety. Qualitative analyses identified four main themes related to the participants' perspectives on digital mental health treatments. Themes centred on participants' openness to trying digital treatments, perceived advantages of digital treatments relative to mainstream face‐to‐face options and motivations for and expectations of seeking digital mental health treatment. Influences on decision‐making included positive beliefs about mental health help‐seeking, previous use of mental health services, digital and mental health literacy and the ability to integrate treatment into their lives. Participants also expressed some unmet decisional needs, such as the desire for information clarifying treatment processes, uncertainty or ambivalence about suitability for self‐guided treatment, and the importance of digital and health literacy. Principal findings are discussed below with reference to the broader research and practical implications.

The current study highlights that several factors influence decisions to take up self‐guided digital treatment for mental health. It is noteworthy that these factors map onto the ‘user‐related’, ‘program‐related’ and ‘technology‐ and environment‐related’ constructs identified within a systematic review of barriers to and facilitators of user engagement with digital mental health interventions, most commonly web‐ and smartphone‐based interventions integrating iCBT and informational or educational resources.^25^ In terms of ‘user‐related’ constructs, positive beliefs about mental health help‐seeking, previous use of mental health services and skills related to digital, mental health and digital health literacy and ability or motivation to integrate the treatment offering into their lives were all cited as influencing factors in participants' treatment decisions. For ‘program‐related’ constructs, the perceived credibility of digital treatment, the extent to which it can be personalised/customised and the level of guidance including nonhuman guidance in the form of structured reminders were again salient factors for participants in the current study. Finally, ‘technology‐ and environment‐related’ constructs emerged in participants' accounts as they considered access costs (especially the low cost of digital treatments compared to traditional face‐to‐face options), the degree of privacy, confidentiality and anonymity afforded by the technology, as well as social influences or perceptions held by family and health professionals. These parallels between influences on decision‐making about uptake, and influences on later ongoing engagement show that digital treatment providers need to acknowledge and address these influences before initial uptake. These findings also underscore the importance of supporting the decision‐making process as a means of promoting later engagement—for instance, using carefully designed decision aids. Indeed, a systematic review to determine the effects of patient decision‐aids on improvements to decisional needs identified some, although mixed, evidence that these decision‐aids led to greater adherence to the patients' chosen option and improved adherence to treatment compared to usual care.^42^

Although participants held overall positive attitudes towards taking up digital mental health treatments, they also reported engaging in limited deliberation around alternative options and cited some unmet decisional needs. For example, participants mentioned wanting to speak to someone within the digital service regarding this treatment option and alternatives before starting treatment, having uncertainty regarding their personality suitability for self‐guided treatment (especially its self‐paced nature) and the prerequisite for adequate digital and health literacy. It is possible that even in the context of self‐guided treatments, initial contact with a therapist to discuss alternative options and personal suitability may be beneficial, especially amongst people with lower levels of digital and/or health literacy. Indeed, a recently published meta‐analysis of digital interventions for depression with varying degrees of contact (e.g., before, during and/or after the intervention) found that contact before and during the intervention exerted independent influences on symptom improvement and treatment engagement.^19^ Further, guided interventions only outperformed unguided interventions when they included contact both before and during the intervention; that is, contact during a guided intervention was not by itself sufficient in producing superior outcomes compared to unguided interventions.^19^ In this sense, there appears to be a crucial role for pretreatment contact with a therapist that occurs synchronously in real time (e.g., defined in this review as a diagnostic interview via phone call, videoconferencing, text chat). Indeed, qualitative research with therapists working within a digital mental health service found that discussions with a therapist over the phone to discuss assessment results and treatment options facilitate informed decision‐making around treatment uptake and guidance.^43^ Even in the absence of a therapist, this pretreatment period still provides an opportune time to provide people with appropriate information and decision‐support resources so that they are clear about treatment processes and the type of treatment that they are receiving.^43^ Having pretreatment information and a clear understanding of treatment are shown to be associated with lower likelihood of people self‐reporting lasting negative effects from treatment,^44^ which may include worsened symptoms, stigma, feelings of hopelessness and failure.^45^

It is noteworthy that the current sample's perceptions of decisional needs were low overall, with average DCS below the average scores reported in other mental health treatment samples (*M* = 32.4 ± 11.4).^46^ Specifically, mean scores for the current sample all fell below 25/100 (range: *M* = 11.40–18.42 for subscales; *M* = 14.47 for total score), which is associated with people implementing a decision as opposed to delaying or being unsure about implementing a decision^38^. These low scores are not unsurprising, given that the current sample perceived limited alternative options and reported engaging in limited deliberation on options. It is possible that some decisional needs within the ODSF^27^, ^28^, ^29^ and thus related DCS are less relevant and applicable to self‐guided digital treatments. Indeed, this study was one of the first, if not the first study, to apply the ODSF to the digital health context, with a recent systematic review identifying no such studies in either mental health or health more generally.^27^ For example, the ‘support’ subscale, which had the lowest mean score in the current sample (*M* = 11.40/100), denotes having enough support and advice from others (e.g., health professionals, family and friends) to make a choice, and making a choice without pressure from others. However, in the context of self‐guided digital treatments, support from others might be less pertinent due to the high level of discretion and privacy in accessing and taking up these treatments. This interpretation is also consistent with participant reports of making a treatment decision independently of others and accords with findings from a study applying the Theory of Planned Behaviour to predict people's intention to engage with digital mental health interventions.^47^ In this quantitative survey‐based study, neither subjective norms nor the perceived social pressure to perform a behaviour emerged as significant predictors of people's intention to continue engaging with a digital mental health intervention.

Alternatively, it could be that current study participants reported lower decisional conflict because they perceived having already made their decision, having completed an initial online assessment and been screened as eligible for treatment (which they were yet to take up at the time). Observed post‐decision decreases in decisional conflict, even without information or decision‐support interventions, have been reported and are said to reflect a psychological need to be comfortable with a decision that has been made.^29^ This said, across all decisional conflict subscales, upper‐range scores (45–66.67/100) were all associated with discontinuation of the chosen option (>37.5/100)^38^. When considering these scores alongside participant comments and the small sample size (*n* = 20), this suggests that at least a proportion of people deciding to take up self‐guided digital treatment for their mental health have unmet decisional needs and require additional decision support. That said, decisional support interventions, such as patient decision‐aids, are likely to be most effective in an earlier decision‐making stage, when people are actively considering their options for treatment.^48^ At this stage, people tend to be more receptive to treatment information, values clarification and decisional guidance.^48^ That said, future research would need to scope out the optimal timing of delivery in the digital mental health context (e.g., deciding between face‐to‐face CBT vs. therapist‐guided iCBT vs. self‐guided iCBT).

## 5 LIMITATIONS

It is important that the current study findings are considered in light of some limitations. First, participants in the current study were taking up self‐guided digital treatment for their mental health in the context of a larger RCT, and as such, their views and experiences may not be representative of people considering this treatment outside a research setting. For example, as part of the RCT protocol, participants received an information statement outlining what treatment involves and were made aware that the clinical research team would be monitoring them for risk or symptom deterioration during treatment and would make direct contact as needed. These features may not be present in all publicly available self‐guided digital treatments and may have given participants the impression that some external support and guidance, although nontherapeutic, were available to them. Further, digital mental health research samples tend to include more older, highly educated participants who are more likely to have accessed mental health treatment previously^18^, ^31^, ^32^, ^33^, ^34^ compared to community‐based cohorts accessing digital mental health services.^22^ That said, all participants self‐referred to the RCT and were not provided with any formal clinical assessment before taking part. This ‘direct to consumer’ model of delivery is consistent with most other self‐guided/unguided digital interventions and therefore, the current findings are likely applicable to these interventions more generally.

Second, as mentioned previously, the interviews were conducted before participants’ commencing treatment but after they completed the initial online assessment and baseline measures and had been screened as eligible. It is possible that the timing of interviews coincided with participants feeling that they had already decided to take up treatment and therefore impacted on their perceptions of decisional needs. Conducting interviews when participants first engaged with the digital clinic's webpage (i.e., before completing an initial assessment and baseline measures) may have elicited more different perceptions of decisional needs but within the broader study protocol, this was not possible.

## 6 CONCLUSIONS

To our knowledge, this study is the first to explore the decision‐making influences, processes and unmet needs among individuals deciding to take up self‐guided digital treatment for depression and anxiety. Some recommendations based on current study insights are that people considering self‐guided digital mental health treatments receive information and decision support that is tailored to their individual preferences and needs. Further, pretreatment interactions and discussions with a clinician (either as part of or separate from the treatment itself, say with a GP) may benefit people's decision‐making about treatment uptake, especially for people with lower digital or health literacy. Finally, providing people with opportunities to assess their readiness and suitability for self‐guided treatments appears crucial, as is presenting evidence‐based digital treatments as a credible and trustworthy option for people with anxiety and depression.

## AUTHOR CONTRIBUTIONS

**Alana Fisher**: Conceptualisation; methodology; data curation; investigation; formal analysis; supervision; funding acquisition; project administration; writing—original draft; writing—review and editing. **Sylvia Eugene Dit Rochesson**: Formal analysis; project administration; writing—review and editing; data curation. **Madelyne A. Bisby**: Conceptualisation; methodology; writing—review and editing; investigation; software. **Amelia J. Scott**: Conceptualisation; methodology; writing—review and editing; investigation. **Milena Gandy**: Conceptualisation; methodology; investigation; writing—review and editing. **Andreea Heriseanu**: Conceptualisation; methodology; investigation; writing—review and editing. **Nick Titov**: Conceptualisation; methodology; software; investigation; writing—review and editing; resources. **Blake Dear**: Conceptualisation; methodology; software; investigation; supervision; resources; writing—review and editing.

## CONFLICT OF INTEREST STATEMENT

The authors declare no conflict of interest.

## Data Availability

Data sharing is not supported by approved HREC submission. Data for this study are not available to share based on current ethics approval.
